# Untargeted metabolomic analysis of the carotenoid-based orange coloration in *Haliotis gigantea* using GC-TOF-MS

**DOI:** 10.1038/s41598-019-51117-9

**Published:** 2019-10-10

**Authors:** Xiaohui Wei, Nan Chen, Bin Tang, Xuan Luo, Weiwei You, Caihuan Ke

**Affiliations:** 1 State Key Laboratory of Marine Environmental Science, Xiamen, 361002 China; 2 College of Ocean and Earth Sciences, Xiamen University, Xiamen, 361002 China; 3 College of the Environment & Ecology, Xiamen University, Xiamen, 361002 China; 4 Fujian Key Laboratory of Genetics and Breeding of Marine Organisms, Xiamen, 361002 China

**Keywords:** Metabolomics, Metabolomics

## Abstract

Seafood coloration is typically considered an indicator of quality and nutritional value by consumers. One such seafood is the Xishi abalone (*Haliotis gigantea*), which displays muscle color polymorphism wherein a small subset of individuals display orange coloration of muscles due to carotenoid enrichment. However, the metabolic basis for carotenoid accumulation has not been thoroughly investigated in marine mollusks. Here, GC-TOF-MS-based untargeted metabolite profiling was used to identify key pathways and metabolites involved in differential carotenoid accumulation in abalones with variable carotenoid contents. Cholesterol was the most statistically significant metabolite that differentiated abalones with orange muscles against those with common white muscles. This observation is likely due to the competitive interactions between cholesterol and carotenoids during cellular absorption. In addition, the accumulation of carotenoids was also related to fatty acid contents. Overall, this study indicates that metabolomics can reflect physiological changes in organisms and provides a useful framework for exploring the mechanisms underlying carotenoid accumulation in abalone types.

## Introduction

Carotenoids are naturally occurring orange and bright-yellow pigments that are found in fruits, vegetables, and certain shellfish and are responsible for coloration in many animals^1,2^. Carotenoids are precursors of vitamin A that accumulate in many animals and aid in immune system function and health improvement^3^. Animals must acquire these pigments through their diets unlike plants, fungi, and bacteria, which can synthesize carotenoids de novo^4,5^. Due to the growing use of carotenoids as antioxidants and the resulting rise in demand for end-use applications, such as in animal feed, foods, and in pharmaceuticals, the carotenoids market is expected to reach $1.7 billion by 2022 (GIA; http://www.strategyr.com/pressMCP-1700.asp). The total carotenoid content in animals can vary, as observed in many marine animals, including fish^6,7^, shrimp^8,9^, and bivalves^10^. Moreover, carotenoid content can vary according to tissue types, seasons, feeding habits, and sex of the individual. Investigations of color polymorphism in marine mollusks currently focus on shell carotenoids^11,12^. In contrast, investigations of coloration in shellfish soft tissue are limited, despite that soft tissues can exhibit high carotenoid contents. For example, the noble scallop (*Chlamys nobilis*) and Yesso scallop (*Patinopecten yessoensis*) contain significantly higher carotenoid contents in their orange adductor muscles than in other tissues. Moreover, orange scallops also have orange mantles and adductor muscles, owing to a high abundance of carotenoids^13,14^.

As in other animals, marine mollusks lack the ability to synthesize carotenoids *de novo* and must acquire them from food sources^13,14^. Consumed carotenoids are then modified through metabolic processing^15^. The accumulation of carotenoids in marine mollusks can only be explained by incorporating an understanding of the biochemical mechanisms associated with carotenoid processing and other underlying processes, including absorption, transport, metabolic transformation, and deposition. Previous studies have attempted to evaluate carotenoid structure, tissue distribution, and the biological function of carotenoids in marine mollusks^16^. In addition, studies at the level of genes, transcripts, and proteins have also elucidated the basis for physiological variation in carotenoid contents in marine mollusks. For example, transcriptional analyses have shown that a novel class B scavenger receptor termed SRB-like-3 is significantly associated with high carotenoid content and the orange coloration of the noble scallop^17^. Moreover, proteomics has revealed that there are seven differentially expressed proteins between a new variety of Yesso scallops and individuals of the common variety^18^. In addition, the two groups exhibit genetic differentiation for those involved in various biological processes, including lipid and glucose metabolism, protein-folding, and protein degradation^18^. Nevertheless, limited information is known about the metabolic mechanisms underlying carotenoid accumulation in marine mollusks, thus necessitating further research.

**Figure 1:**
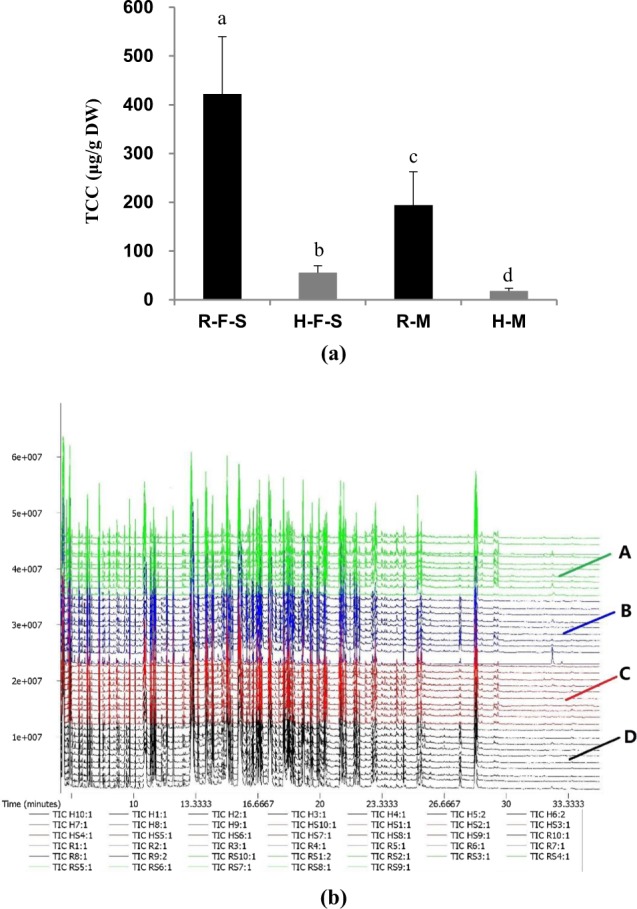
(**a**) The total carotenoid content (TCC) in different muscle tissues of orange and common abalones (μg/g DW), where orange-foot muscle (R-F-S), orange-adductor muscle (R-M), common-foot muscle (H-F-S), and common-adductor muscle (H-M) represents. (**b**) GC-TOF-MS total ion chromatograms (TICs) of different muscle parts of orange and common abalone extracts: (A) R-F-S, (B) R-M, (C) H-F-S, (D) H-M.

The Xishi abalone (*Haliotis gigantea)* is naturally distributed along coastal areas of Japan but it was introduced to China in 2003^19^. The previous study has shown that the yellow-orange foot in abalones was related to carotenoid pigments^20^. Abalones display conspicuous differences in muscle color that can either be the common coloration or orange coloration due to carotenoid enrichment. And the total carotenoid contents of muscle were significantly higher (P < 0.01) in orange abalones compared to common abalones (Fig. 1a). Thus, the carotenoid-based orange coloration of some *H. gigantea* muscles provides a useful framework for investigating the mechanisms of carotenoid accumulation in marine mollusks.

Metabolomics is a recently developed technique that has been widely used to investigate the metabolic profiles of organisms. In particular, metabolomics can help evaluate sources of variation from genetic or environmental origins^21^. Metabolites are the end products of cellular regulatory processes and can be regarded as the final response of biological systems to genetic or environmental changes^22^. Consequently, genetic differences are ultimately implicated by metabolic profile differences among organisms. ^1^H-NMR spectroscopy, in conjunction with pattern recognition methods, has been previously used to investigate the responses of *H. diversicolor* muscles and gills to thermal and hypoxic stresses^23^. Identifying the specific metabolite differences associated with differential carotenoid accumulation can also provide a more complete picture of the physiological state of individuals during this process^24^. For example, metabolomics revealed the presence of five phytohormonal metabolites that significantly influence carotenoid metabolisms in tomato fruits^25^. Likewise, an integrated analysis of the transcriptome and metabolome of three colored potato cultivars provided evidence for a regulatory system that is involved in the pigmentation of germinated sprouts^26^.

In the present study, GC-TOF-MS-based (Gas Chromatography Time-Of-Flight Mass Spectrometry) untargeted metabolite profiling was used to compare the metabolomic differences of abalones exhibiting different carotenoid contents. The overarching goal of this research was to identify key pathways and crucial metabolites that are associated with differential carotenoid accumulation capabilities that could then inform our understanding of carotenoid accumulation mechanisms in abalones.

## Results

### Total carotenoid contents (TCC) of muscles

The TCC of abalone muscles ranged from 10.15 to 602.66 μg/g dry weight (DW). A one-way ANOVA indicated that TCC was significantly different (*P* < 0.05) among individuals with different coloration and also among tissue types. The TCC of foot and adductor muscles were significantly higher (*P* < 0.01) in orange abalones compared to common abalones. In addition, the TCC of foot muscles were significantly higher (*P* < 0.05) than in adductor muscles (Fig. 1a).

### Metabolomic analysis

Untargeted metabolite profiling was used to determine metabolic differences between abalones with different muscle colors. The total ion current chromatograms from GC-TOF-MS analysis are shown for the 40 samples in (Fig. 1b). More than 452 compounds were identified among all samples, including fatty acids and their conjugates, amino acids, peptides, pterins and their derivatives, phenylpyruvic acid derivatives, saccharides, flavonoids, and other substances (Table S1).

**Figure 2:**
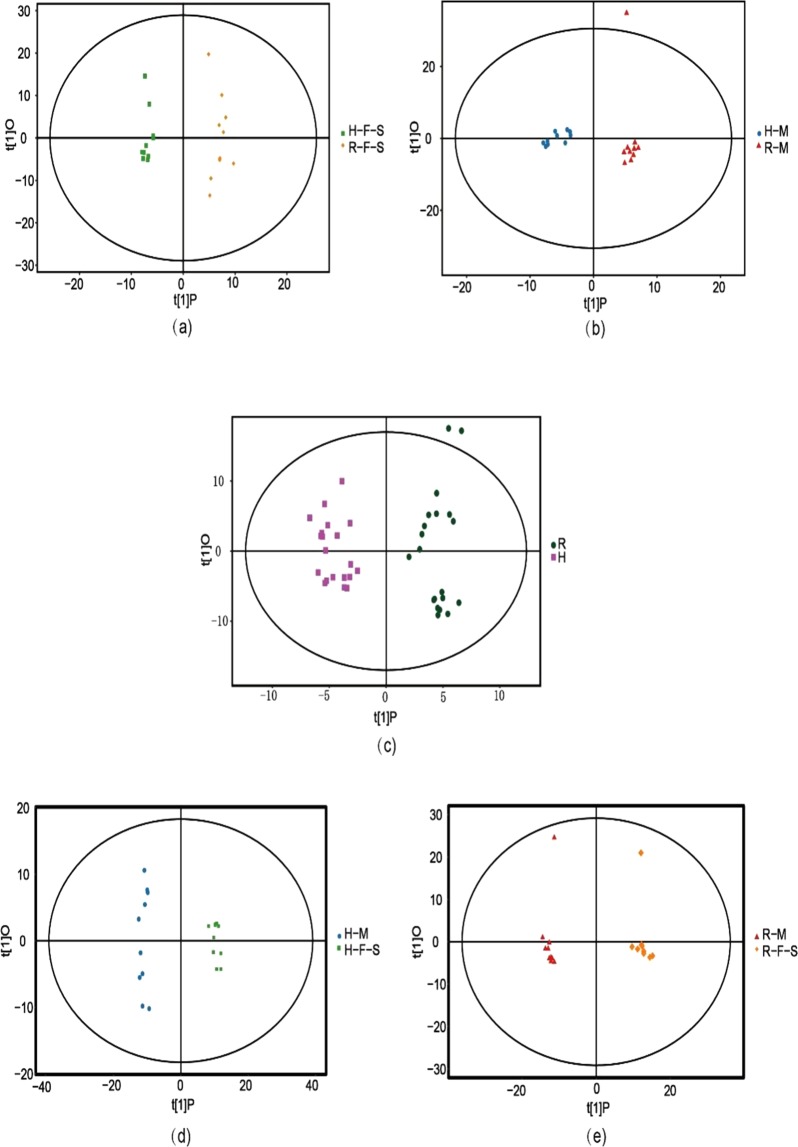
Score scatter plot of OPLS-DA model for different pairwise comparisons: (**a**) H-F-S vs. R-F-S, (**b**) H-M vs. R-M, (**c**) H vs. R, (**d**) H-F-S vs. H-M, and (**e**) R-F-S vs. R-M.

Orthogonal projections to latent structures-discriminate analysis (OPLS-DA) were used to evaluate metabolic profile changes among different sample groups. In particular, pairwise comparisons were performed for the H-F-S vs. R-F-S, H-M vs R-M, H vs R groups, H-F-S vs H-M, and R-F-S vs R-M groups. The validation parameters for the five OPLS-DA comparisons were R^2^Y = 0.974 and Q^2^ = 0.766 for H-F-S vs. R-F-S, R^2^Y = 0.957 and Q^2^ = 0.596 for H-M vs. R-M, R^2^Y = 0.942 and Q^2^ = 0.71 for H vs. R, R^2^Y = 0.992 and Q^2^ = 0.938 for H-F-S vs. H-M, and R^2^Y = 0.989 and Q^2^ = 0.928 for R-F-S vs. R-M (Table S2). Clear separations between groups were observed in the OPLS-DA score plots and most samples plotted in the 99% confidence interval (Hotelling’s T-squared ellipse), indicating marked differences in the spectral characteristics of the groups (Fig. 2). In addition, the R^2^Y value of the model was close to 1, indicating accurate approximation of the data by the model. Taken together, these results indicated that the model generally explained the differences between the two sample groups well (Fig. S1).

### Differential metabolomic profiling

**Figure 3:**
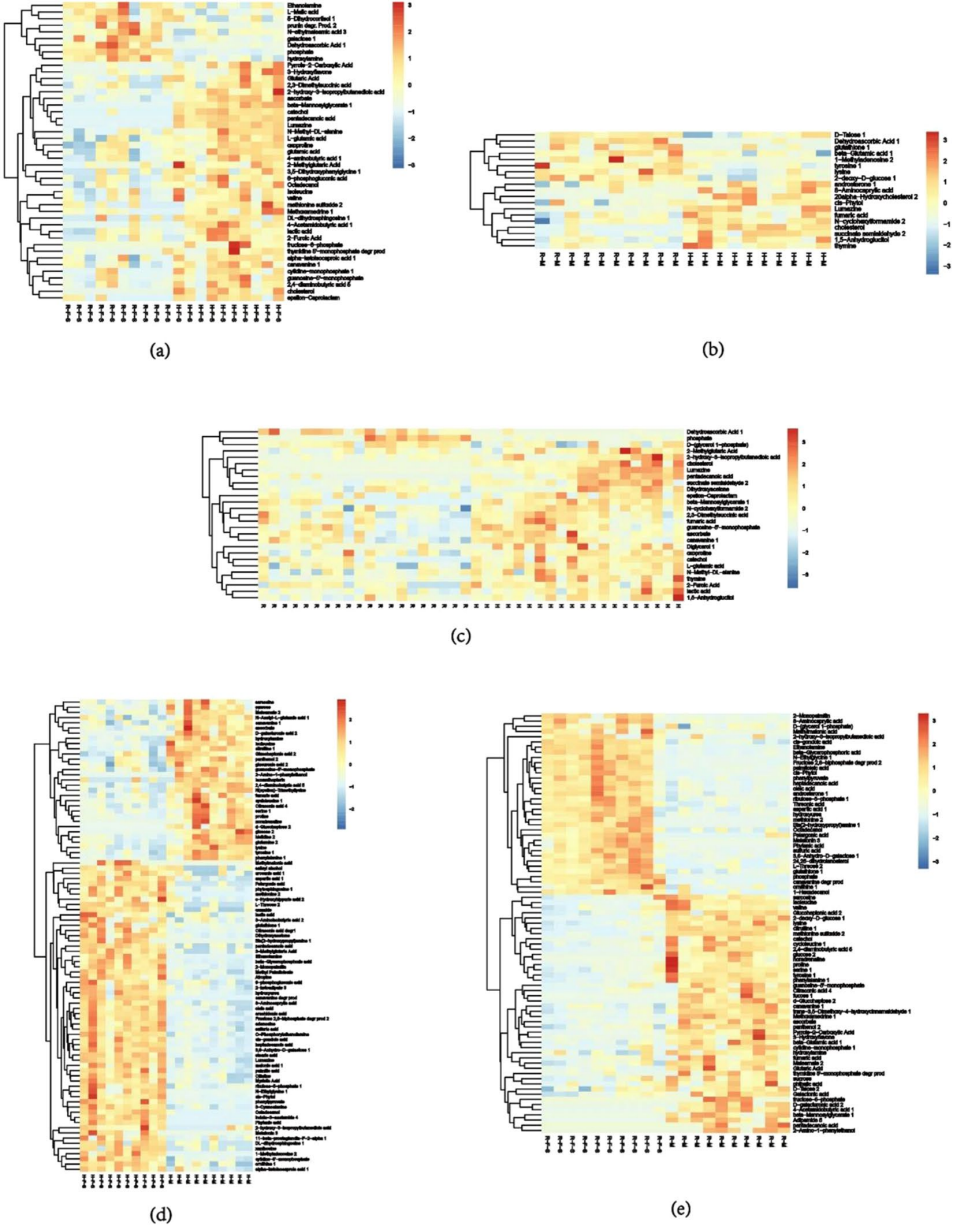
Heatmaps showing hierarchical clustering of metabolite profiles for different groups: (**a**) H-F-S and R-F-S, (**b**) H-M and R-M, (**c**) H and R, (**d**) H-F-S and H-M, and (**e**) R-F-S and R-M. Warm colors represent higher compound contents, whereas cool colors represent lower compound contents.

The variable importance in the projection (VIP) value can be used as a criterion to identify important explanatory variables in OPLS models. Metabolites were considered significantly different among groups when the VIP > 1 and the *P* < 0.05 based on a Student’s *t* test. Metabolites that were significantly different between groups are provided in Table S3. Clustering analysis based on unsupervised hierarchical clustering of samples (Fig. 3) indicated clear separation of samples based on metabolite differences among the five groups. A total of 78 significantly different metabolite abundances were identified between the H-F-S and R-F-S groups. Of these, 19 metabolites increased in abundance, while 59 decreased in the R-F-S tissues relative to those of the H-F-S group. Forty-six of these metabolites could be annotated, and these included cholestane steroids, fatty acids and their conjugates, amino acids, and peptides (Fig. 3a). A total of 47 metabolites with differential abundances were observed between the H-M and R-M group tissues. Among these, 17 metabolites increased in abundance while 30 decreased in abundance in the R-M group tissues relative to those of the H-M group. Nineteen of the differential metabolites were annotated and these mainly included cholestane steroids, amino acids, peptides, and monosaccharides (Fig. 3b). A total of 49 metabolites were differentially abundant between the R and H group tissues. Among these, six metabolite abundances increased and 43 decreased in the R group tissues relative to those of the H group. Of the 49 differentially abundant metabolites, 27 were identified (Fig. 3c). Especially, three metabolites were found both in the H-F-S vs R-F-S, H-M vs R-M and H vs R groups.

A total of 171 differential metabolites were detected between the H-F-S and H-M groups. Of these, 104 metabolites increased and 67 metabolites decreased in the tissues of the H-F-S group relative to the H-M group. These metabolites included fatty acids and conjugates, amino acids, carbohydrate, peptides, pterins and their derivatives, and phenylpyruvic acid derivatives (Fig. 3d). Of the 145 metabolites with differential abundances between the R-F-S and R-M groups, 60 increased and 85 decreased in the tissues of the R-F-S group relative to those of the R-M group. Eighty-one of these metabolites could be annotated, and these primarily included fatty acids and their conjugates, amino acids, carbohydrate, peptides and their derivatives (Fig. 3e).

### Characterization of differential pathways

**Figure 4:**
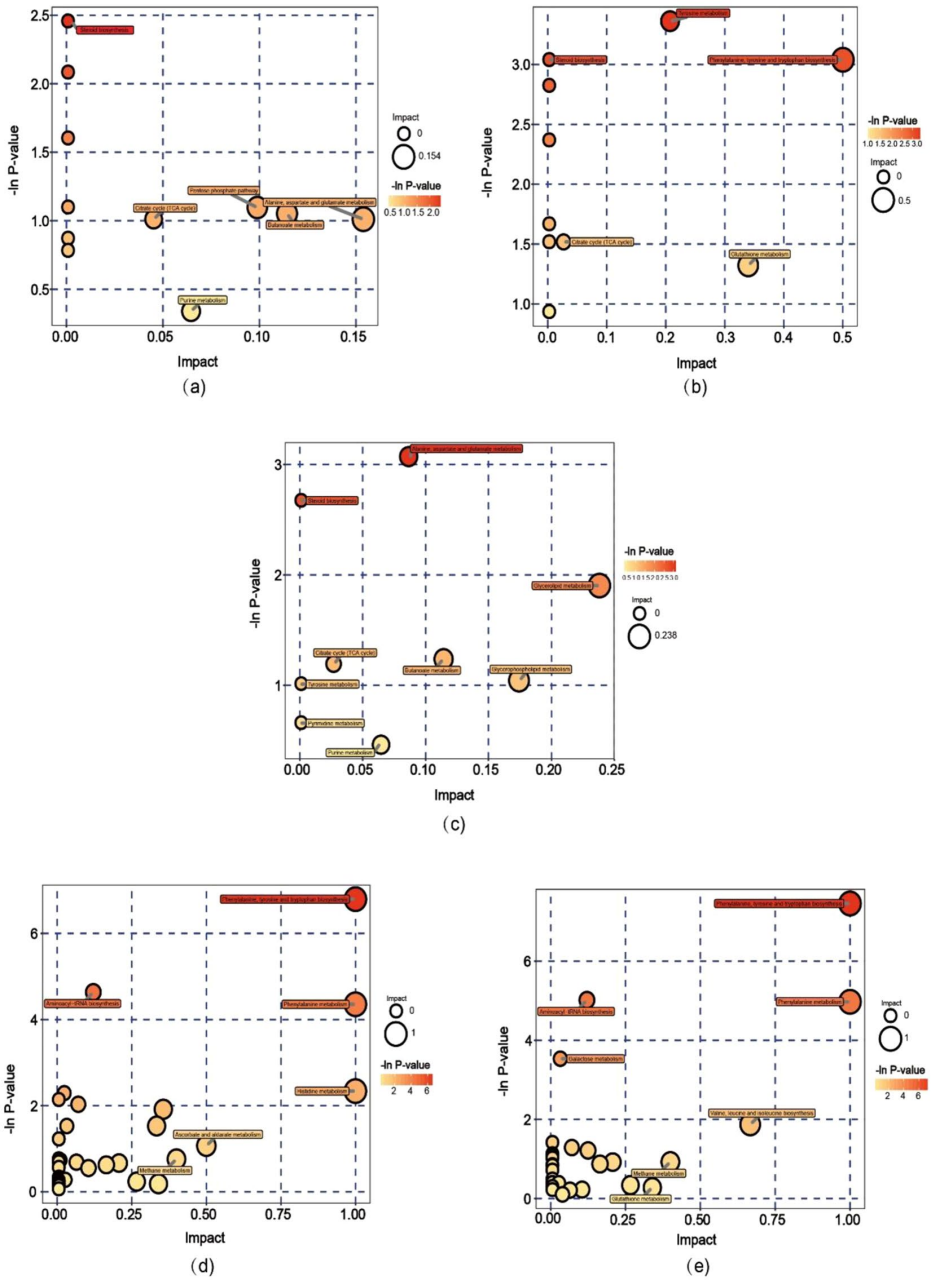
Pathway analysis bubble plots showing differences between comparisons for: (**a**) H-F-S and R-F-S, (**b**) H-M and R-M, (**c**) H and R, (**d**) H-F-S and H-M, and (**e**) R-F-S and R-M.

A total of 11 differential metabolite pathways were detected in the R-F-S and H-F-S comparison. These pathways included steroid biosynthesis; alanine, aspartate and glutamate metabolism, and butanoate metabolism (Table S4). In particular, pathway analysis indicated that alanine, aspartate, and glutamate metabolism most differentiated R-F-S and H-F-S profiles and exhibited the biggest impact factor (Fig. 4a, Table 1). Only 10 differential metabolite pathways were detected in the R-M and H-M comparison. These included tyrosine metabolism; steroid biosynthesis; phenylalanine, tyrosine, and tryptophan biosynthesis; and phenylalanine metabolism (Table S4). Pathway analysis indicated that tyrosine metabolism most segregated the R-M and H-M group profiles and was most statistically significant. In addition, phenylalanine, tyrosine, and tryptophan biosynthesis exhibited the biggest impact factor (Fig. 4b, Table 1). Nine differential metabolite pathways were observed between the R and H group profiles and these included alanine, aspartate, and glutamate metabolism; steroid biosynthesis; and tyrosine metabolism (Table S4). Among these, alanine, aspartate, and glutamate metabolism was most statistically significant by pathway analysis, indicating its importance in segregating these two groups (Fig. 4c, Table 1). Three pathways were observed to differentiate all three pairs of groups, which included steroid biosynthesis; alanine, aspartate, and glutamate metabolism; and the citrate cycle (TCA cycle). Specifically, cholesterols involved in steroid biosynthesis both decreased in the R-F-S vs H-F-S, the R-M vs H-M, and the R vs H comparisons. Consequently, these results suggest that different cholesterol content may be related to differences in carotenoid contents. Thirty-six differential metabolite pathways were identified in the H-F-S and H-M comparisons, while 27 were observed between the R-F-S and R-M group profiles (Table S4). Enrichment analysis and topological differences in pathways indicated that phenylalanine, tyrosine, and tryptophan biosynthesis most significantly differentiated the H-F-S and H-M groups as well as the R-F-S and R-M groups, which shared similar carotenoid content differences (Fig. 4d,e, Table 1).

**Table 1:** Results of further analysis of different metabolite’s pathway in the different groups.

Group	Pathway	Metabolites	Raw p	−log(p)	Impact
R-F-S and H-F-S	Alanine, aspartate and glutamate metabolism	L-glutamic acid	0.36382	1.0111	0.15385
R-M and H-M	Tyrosine metabolism	tyrosine	0.034675	3.3617	0.2073
	Phenylalanine, tyrosine and tryptophan biosynthesis	tyrosine	0.047869	3.0393	0.5
	Steroid biosynthesis	cholesterol	0.047869	3.0393	0
R and H	Alanine, aspartate and glutamate metabolism	succinic acid semialdehyde fumaric acid	0.046361	3.0713	0.08654
	Steroid biosynthesis	cholesterol	0.069051	2.6729	0
H-F-S and H-M	Phenylalanine, tyrosine and tryptophan biosynthesis	tyrosine phenylalanine phenylpyruvate	0.001117	6.7976	1
R-F-S and R-M	Phenylalanine, tyrosine and tryptophan biosynthesis	tyrosine phenylalanine phenylpyruvate	0.000578	7.4567	1

Raw p: represents P value of enrichment analysis of metabolic pathway; −log(p): the negative common logarithm of P value; Impact: the Impact value of topology analysis of metabolic pathway.

### Identification of crucial metabolites with multivariate data analysis

**Figure 5:**
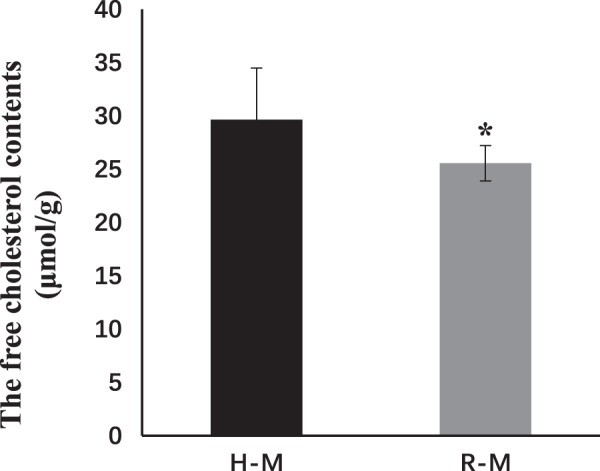
Comparison of cholesterol abundances between H-M and R-M based on a Free Cholestenone Content Assay Kit. *P < 0.05.

OPLS-DA and analysis of the corresponding loading plots were used to identify differences among samples and detect key differential metabolites among groups (Figs 2, S2). The metabolites that were differentially represented among groups are provided in Table 2. A total of 46 metabolite abundances were significantly different between the profiles of the R-F-S and H-F-S groups while 19 were identified between the R-M and H-M profiles. These metabolites primarily included cholestane steroids, amino acids, and peptides (Fig. 3). Higher dehydroascorbic acid levels were detected in both the R-F-S and R-M group profiles compared to those of the H-F-S and H-M groups. In addition, tyrosine levels were higher in the R-M profiles than in those of the H-M group. In addition, cholesterol and lumazine levels were significantly lower in the R-F-S and R-M profiles compared to those of the H-F-S and H-M groups (Figs S2a,b, 3a,b). Increased phosphate, D-(glycerol 1-phosphate), and dehydroascorbic acid levels were observed in the R group profiles relative to those of the H group. In contrast, cholesterol, lumazine, fumaric acid, ascorbate, lactic acid, and other compounds exhibited reduced abundances in the R group profiles compared to those for the H group (Figs S2c, 3c). Cholesterol was the most statistically significant differential metabolite associated with differences in carotenoid contents in the R-F-S and H-F-S, R-M and H-M, and R and H comparisons. In general, cholesterol levels were lower in orange abalone tissues relative to common abalone tissues (Fig. S2a–c). Indeed, cholesterol was the most important metabolite differentiating the R and R-M groups from the H and H-M groups, as evinced by the highest VIP value, and clearly lower abundance (*P* < 0.001) in the R and R-M group profiles. The differences in cholesterol content between the R-M and H-M groups were further validated using a Free Cholestenone Content Assay Kit (Fig. 5).

**Table 2:** The change of important different metabolites among different groups.

Metabolites	VIP	P-Value	Fold Change	R-F-S to H-F-S	R-M to H-M	R to H	H-F-S to H-M	R-F-S to R-M
Stearic acid	1.8	1.62E-06	2.36	—	—	—	↑	—
Oleic acid	1.73	3.16E-07	2.27	—	—	—	↑	—
	1.82	4.97E-05	2.35	—	—	—	—	↑
Palmitoleic acid	1.30	0.000118	14.74	—	—	—	—	↑
Arachidonic acid	1.8	1.24E-08	3.34	—	—	—	↑	—
Palmitic acid	1.7	5.96E-05	1.97	—	—	—	↑	—
Myristic Acid	1.67	3.69E-06	1.87	—	—	—	↑	—
Tyrosine 1	1.67	1.17E-06	0.51	—	—	—	↓	—
	1.90	3.39E-07	0.32	—	—	—	—	↓
	1.91	1.08E-02	1.33	—	↑	—	—	—
Phenylalanine 1	1.57	0.000101	0.40	—	—	—	↓	—
	1.92	1.90E-05	0.29	—	—	—	—	↓
Phenylpyruvate	1.40	9.90E-05	12.28	—	—	—	↑	—
	1.43	0.000149	11.74	—	—	—	—	↑
Cholesterol	2.39	0.001079	1.23E-07	↓	—	—	—	—
	3.04	8.56E-07	0.08	—	↓	—	—	—
	3.61	3.90E-05	0.02	—	—	↓	—	—

↑, increase; ↓, decrease; —, no change. VIP: the value of variable importance in the projection; P-VALUE: P-value of Student’s t-test; FOLD CHANGE: the ratio of the contents of experimental substances in two groups.

Tyrosine, phenylalanine, phenylpyruvate, and fatty acid were predominantly differential metabolites in the H-F-S vs H-M and R-F-S vs R-M comparisons. Tyrosine and phenylalanine levels were lower in the H-F-S and R-H-S group profiles compared to those of the H-M and R-M groups, while phenylpyruvate levels were higher in the former. Stearic acid, oleic acid, palmitoleic acid, and other fatty acid abundances were elevated in the H-F-S group relative to the H-M group (Figs S2d, 3d, Table 2). In addition, oleic acid and palmitoleic acid abundances were both elevated in the R-F-S group profiles relative to the R-M group (Fig. 3e, Table 2). Besides, we also detected the lower glucose and sucrose contents in H-F-S and R-F-S groups compared to those of the H-M and R-M groups (Fig. 3d,e).

## Discussion

The analysis of organismal metabolomics via GC-MS has been widely used in recent years to evaluate the metabolic state of various biological models. The first metabolomic study analyzed the metabolic profile of urine and tissue extracts using GC-MS^27^. Metabolomic profiling in concert with gas chromatography has also been coupled to time-of-flight mass analyzers (GC-TOF-MS) to measure changes in endogenous metabolites of a rat model and identify potential biomarkers of estrogen-deficiency-induced obesity^28^. In mollusk models, the application of integrated transcriptomics and GC-TOF-MS-based metabolomics provided insight into the regulation of glycogen content within the pacific oyster *Crassostrea gigas*^29^. Of importance to the present study, the metabolomics involved in carotenoid metabolism have been recently investigated in several studies. For example, a GC-MS-based metabolomics approach was used to investigate glycerol metabolism and the mechanisms of carotenoid production in three strains of *Rhodosporidium toruloides* during different growth phases^30^. In addition, GC-MS-based metabolomics and multivariate analysis have been used to evaluate the potential use of arachidonic acid treatment of *Blakeslea trispora* to enhance production of β-carotene by investigating intracellular biochemical changes at different time points after arachidonic acid treatment^31^. GC-MS is one of the most mature chromatography-mass spectrometry techniques and features several advantages including high resolution, high sensitivity, good reproducibility with a large number of standard metabolite spectrum libraries, and relatively low costs. Moreover, GC-MS can resolve metabolites with small relative molecular weights and polarity, low boiling points, or volatile substances after derivatization. In the present study, GC-TOF-MS was used to identify metabolites that differed between the muscle tissues of orange and common abalones that displayed significantly different carotenoid contents. The overall goal of the investigation was to identity key pathways and crucial metabolites that differentiated these groups of abalones with respect to carotenoid production.

Metabolomics can provide a high resolution overview of metabolic pathways and physiological states that are affected during various processes. Activities of key metabolic pathways significantly differed between groups analyzed in this study. In particular, the alanine, aspartate, and glutamate metabolism pathway significantly differed by pathway analysis when comparing the R vs H profiles and the R-F-S vs H-F-S profiles. Besides, tyrosine metabolism and phenylalanine, tyrosine, and tryptophan biosynthesis were important for differentiating the profiles of the R-M and H-M groups. The steroid biosynthesis pathway was important for segregating groups for all three comparisons of the R vs H, R-F-S vs H-F-S, and R-M vs H-M groups. Lastly, the phenylalanine, tyrosine, and tryptophan biosynthesis pathway was the most important pathway differentiating the profiles of the H-F-S and H-M groups, in addition to the R-F-S and R-M groups. Cholesterol, which is involved in steroid biosynthesis, exhibited differential abundances in the R vs H, R-F-S vs H-F-S, and R-M vs H-M comparisons. Thus, cholesterol content may be related to differences in carotenoid content.

Cholesterol is an important component of eukaryotic cell membranes that influences the biophysical properties of cell membranes and serves as a precursor for vitamin D, bile acids, and steroid hormones^32^. Liposomes are the most common route for incorporation of cholesterol into membranes, wherein liposomes with different carrier polarities are utilized^33^. Carotenoids are a large family of lipophilic compounds that can be synthesized in the membranes of plants and microorganisms, but they are minor constituents of animal membranes^34^. In animals, carotenoids are generally absorbed by the small intestinal epithelium after being incorporated into mixed micelles that form by the detergent action of bile salts and the hydrolysis of emulsified lipids (triacylglycerols) by pancreatic lipases (PLs) and colipase^35^. Consequently, the absorption of carotenoids and cholesterol are highly dependent on lipid abundances. The interference and modulatory effects of cholesterol and carotenoids in membranes have been previously reported, in addition to their localization in lipid aggregates^36,37^. In particular, cholesterol has been shown to change the membrane order state and broaden or even completely suppress the phase transition peak of liposomes. Further, cholesterol can affect the permeability to ions, compressibility of bilayers, and the diffusion of oxygen across membranes^38,39^. Likewise, carotenoids that are incorporated into liposomes will also modulate membrane hydrophobicity, microviscosity, permeability to ions, and the diffusion of oxygen, while also protecting phospholipids against oxidation^37^.

The insertion of cholesterol together with carotenoids into different membranes is selective and depends on membrane fluidity, carotenoid polarity, and the cholesterol to carotenoid ratio^40^. When carotenoids and cholesterols incorporate into liposomes together, carotenoid incorporation is strongly reduced. Indeed, carotenoids can only be effectively incorporated into membranes when membranes exhibit low cholesterol concentrations^40^. Further, a high level of competition has been observed for cholesterol and carotenoid incorporation into liposomes^41^. Specifically, carotenoid competes strongly with cholesterol for incorporation into cholesterol-rich membranes. Thus, cholesterol can act as a barrier for the absorption of carotenoids, depending on their polarity^42^. Here, abundances of cholesterols involved in steroid biosynthesis were lower in the muscles of orange abalones compared to those of the common variety. In addition, cholesterol exhibited the most statistically significant association with carotenoid contents when comparing the metabolite profiles of the R and H groups. Nevertheless, similar patterns in cholesterol differences have not been observed in other aquatic animals. Significant differences in fatty acid levels were observed between the adductors of orange and common Yesso scallops, but cholesterol levels did not differ between the two groups^43^. In contrast, there were no significant differences in the fatty acid contents between orange and common abalones analyzed here. This suggests that differences in carotenoid content in the foot and adductor muscles of Yesso scallops was primarily associated with fatty acids, but that differences in the carotenoid content of muscles between orange and common abalones are associated with cholesterol abundances. Different metabolic pathways may underlie these observed differences, which requires further investigation.

In addition to the above observations, we also observed higher tyrosine levels in the muscles of the R-M group compared to those of the H-M group. Increased tyrosine content in the muscles of the orange abalone may be due to a blockage of melanin biosynthesis. The reduced ability to synthesize melanin in conjunction with increased carotenoid content may contribute to the bright orange coloration of these individuals. Nevertheless, the precise mechanisms underlying orange coloration should be further explored.

Carotenoid contents were markedly higher in foot muscles than in adductor muscles in the abalones analyzed here. The key biosynthetic pathway that differentiated the H-F-S vs. H-M and R-F-S vs. R-M groups was the biosynthesis of phenylalanine, tyrosine, and tryptophan. Accordingly, tyrosine and phenylalanine levels were lower in the H-F-S and R-F-S group tissues compared to those of the H-M and R-M groups, while phenylpyruvate levels were higher in the former. Tyrosine and phenylalanine are aromatic amino acids and are also essential amino acids for protein synthesis. In addition, they are precursors in the biosynthesis of many neurotransmitters, including serotonin, L-dopa, dopamine, norepinephrine, and epinephrine^44^. Phenylalanine is hydroxylated to tyrosine by phenylalanine hydroxylase (PAH), which is the only limiting step for the metabolism of phenylalanine in mammals^45^. Most phenylalanine will be hydroxylated to tyrosine that is then used in the synthesis of other important neurotransmitters and hormones that participate in sugar and fat metabolisms. If this process is obstructed, phenylalanine can be metabolized by other mechanisms to produce phenylpyruvate or will otherwise accumulate within individuals. Tyrosine is commonly considered to be the substrate for the synthesis of melanin, which contributes to the darkening of animal skins, imparting black, brown, and sometimes yellow coloration.

The coloration of aquatic animals is mainly determined by carotenoids, melanin, guanine, and other pigments, wherein the final coloration of organisms is due to the combined effect of many kinds of pigments. The decreases in phenylalanine content in the H-F-S vs. H-M and R-F-S vs. R-M comparisons leads to lower tyrosine levels that then decreases melanin formation, thereby resulting in a dominance of carotenoids among the coloration pigments. The concomitant increase in phenylpyruvate further indicates that the conversion of phenylalanine to tyrosine may be blocked in these organisms. It is possible that changes in phenylalanine hydroxylase synthesis contribute to the above observations, but this hypothesis should be investigated further.

Carotenoids are lipophilic molecules that are intimately involved in fat absorption, transport, and metabolism. The fat-soluble properties of carotenoids combined with the absorption of dietary lipids results in increased lipid contents of foods, which also benefits carotenoid absorption^46^. Fatty acids exhibit significantly different abundances in the adductors of orange scallops compared to common scallops, as noted by significant increases in fatty acid contents, and especially oleic and linoleic acids^43^. Specifically, stearic, palmitic, nutmeg, oleic, acid, and myristic acid contents were significantly higher in foot muscles compared to adductor muscles of common abalones. In addition, oleic and palmitic acid contents were higher in the foot muscles of orange abalones relative to their adductor muscles (Table 2). Free fatty acids, and especially oleic acid, can promote the effective absorption of beta-carotene^47^. Thus, it is apparent that the combined transport of unsaturated fatty acids and carotenoids affects the absorption and transport of carotenoids in animals^48,49^.

The results indicated that differentiation between foot muscles and adductor muscles with various carotenoid contents were predominantly related to amino acid metabolism and fatty acid metabolites. However, several carbohydrate metabolites were also significantly altered. According to the results, we detected the lower glucose, sucrose contents in H-F-S and R-F-S groups compared to those of the H-M and R-M groups. Carotenoid from natural sources have become increasingly important as therapeutic agents against oxidative stress and inflammation-related diseases such as diabetes. It has also been shown that an abnormal elevation in the blood glucose level will lead to the development of diabetes, which is characterized by hyperglycemia, that also is associated with oxidative stress^50^. Some researches reported that the supplementation of carotenoid significantly reduced diabetic plasma glucose level^51,52^. In our study, we detected the lower glucose level in the adductor muscles with the higher carotenoid content compared to the foot muscles in abalones. Meanwhile, we also found that the content of sucrose was decreased, which may supplement the lack of glucose to ensure the normal metabolic process in abalones during standard farming conditions. However, it was not clear whether changes in the carotenoid content affected the above observations, which need to be further explored in the future.

In conclusion, GC-TOF-MS-based metabolomics was used to accurately estimate the physiological status associated with differential carotenoid accumulation in abalones. Cholesterol was the most statistically significant metabolite that differentiated abalones with orange and common muscles. However, similar observations have not been observed in other aquatic animals. We hypothesize that cholesterol was significantly lower in the orange muscle tissues of abalones due to the competitive relationship between cholesterols and carotenoids during cellular absorption. Fatty acids also likely affected the accumulation of carotenoids. In addition, changes in tyrosine, phenylalanine, and phenylpyruvate levels between abalones with different colored muscles indicate that the color differences of the abalones’ foot and adductor muscles may be a result of the combined effects of melanin and carotenoid. In summary, these results demonstrate that metabolomics can be used to assess the physiological variation and detailed metabolite profiles of aquatic organisms. In addition, these data provide a framework for understanding carotenoid accumulation mechanisms in different types of abalones, and they also establish a foundation for the cultivation of new varieties of abalones that are enriched in carotenoids.

## Materials and Methods

### Animal treatment

Orange-colored (group R, 64.31 ± 5.07 mm shell height) and common (group H, 63.14 ± 5.97 mm shell height) *H. gigantea* individuals were collected from the Fuda Abalone Factory in the Fujian Province. Approximately 16 abalones for each group were maintained in an aerated aquarium containing 20 L of filtered, recirculating seawater maintained at 22 ± 1 °C. Abalones were fed *Gracilaria lemaneiformis* for one week prior to sampling.

### Sample preparation

**Figure 6:**
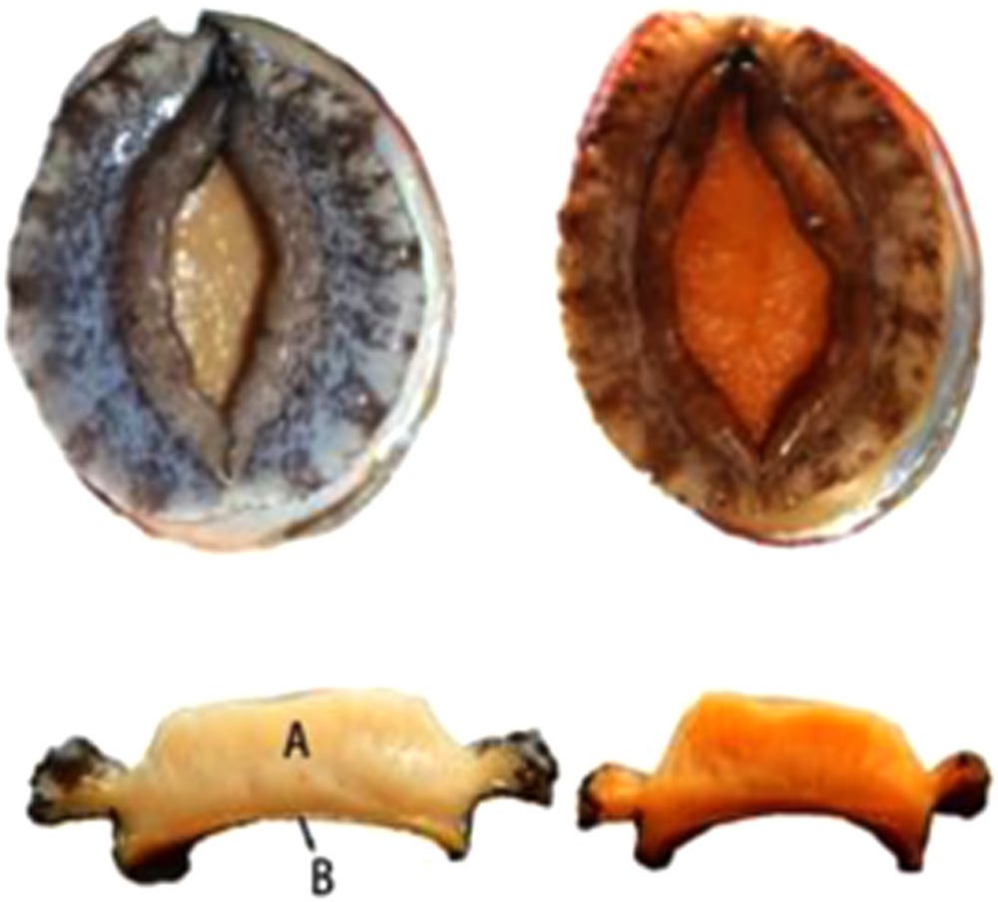
Abalones with common (left) and orange (right) muscles (top). Cross-sectional view of abalone muscles (bottom), with labeling of adductor (A) and foot (B) muscles.

After the acclimation period, the muscle tissues from ten individuals within each group were dissected, divided into adductor and foot muscle sections and then immediately frozen with liquid nitrogen and stored at −80 °C for later GC-TOF-MS analysis (Fig. 6). The comparative groups were defined as: orange-foot muscle (R-F-S), orange-adductor muscle (R-M), common-foot muscle (H-F-S), and common-adductor muscle (H-M). The R group represents the combination of the R-M with R-F-S subgroups, while the H group represents the combination of the H-M with H-F-S subgroups.

Tissue samples were placed in 2-ml Eppendorf tubes and extracted using a mixture of 0.48 ml solvent (methanol: chloroform = 3:1 v/v) and an internal standard established using 24 μl of L-2-chlorophenylalanine (1 mg/ml stock in dH_2_O). The samples were then mixed for 30 s. A ball mill was used to homogenize extracts for 4 min at 45 Hz, followed by ultrasound treatment for 5 min while incubating in ice water. The process was repeated and the resultant mixture was then centrifuged for 15 min at 13,000 rpm and 4 °C. Supernatant (0.4 ml) was transferred to a new 2 ml GC/MS glass vial, and 10 μl was taken from each sample and pooled as a quality control (QC) sample. Samples were dried in a vacuum concentrator without heating after adding 80 μl of methoxy amine hydrochloride (20 mg/ml in pyridine), and then incubated for 30 min at 80 °C. BSTFA (Bis(trimethylsilyl) Trifluoroacetamide) reagent (1% TMCS: Trimethylchlorosilane, v/v) was added (100 μl) to the sample aliquots, and the QC samples were then incubated at 70 °C for 1.5 h. Then, 8 μl of FAMEs (Fatty Acid Methyl Ester) was added to samples, and they were cooled to room temperature and mixed thoroughly for GC-MS analysis.

### GC-TOF-MS analysis

An Agilent 7890 gas chromatograph system equipped with a Pegasus HT time-of-flight mass spectrometer was used to perform GC-TOF-MS analysis. The 1 μl analyte was analyzed using the splitless mode. The carrier gas was Helium, and 3 ml min^−1^ was set as the front inlet purge flow, and 1 ml min^−1^ was set as the gas flow rate through the column. The initial temperature was maintained at 50 °C for 1 min, followed by increase at a rate of 10 °C min^−1^, until reaching 310 °C, when it was maintained for 8 min. Injection temperature was 280 °C, the transfer line temperature was 270 °C, and the ion sources temperature was 220 °C. The energy of electron impact mode was −70 eV. Mass spectrometry data were generated in full-scan mode using an m/z range of 50–500, with 20 spectra per second following a 366-s solvent delay.

### Total carotenoid content (TCC) measurement

Total carotenoid contents were determined from the adductor (A) and foot (B) components of muscle tissues (Fig. 6) from six abalones within each group. The samples were dried using a vacuum freeze-dryer and then ground to fine powders in mortars. Total carotenoids were extracted using previously described methods^10^. Homogenized samples (0.2 g) were then added to 7 ml of acetone and shaken at 200 rpm/min for 2 h in the dark at room temperature. The process was repeated twice, and the extraction was then centrifuged at 3,000 rpm for 5 min. The resultant supernatant was scanned using a UV–vis recording spectrophotometer (UV2501PC, Japan) in the wavelength range of 400 to 700 nm. The absorption value at 480 nm was then used to calculate the TCC with an extinction coefficient E (1%, 1 cm) of 1.900^10^.

### Free cholesterol (FC) content measurement

FC contents were measured from the adductor (A) component of muscle tissues (Fig. 6) from six abalones within each group using a Free Cholestenone Content Assay Kit (BC1890) provided by Beijing Solarbio Science & Technology Co., Ltd., China. Homogenized samples (0.1 g) were added to 1 ml of isopropyl alcohol to extract free cholesterol. The extraction was then centrifuged at 8,000 g for 10 min at 4 °C. Homogenized samples were then added to the FC Assay Buffer for incubation (5 min) and scanned in a multimode plate reader (Tecan Infinite M200 Pro, Switzerland) at 500 nm. The standard was used to determine the free cholesterol contents.

### Multivariate and statistical analyses

The Chroma TOF 4.3X software package (LECO Corporation, Saint Joseph MI, USA) and the LECO-Fiehn Rtx5 database were used to extract raw peaks, filter and calibrate data baselines, align peaks, deconvolution analysis, peak identification, and integration of peak areas^53^. Peak identification was conducted using the RI (retention time index) method with an RI tolerance of 5,000. Metabolic features were removed when detected in < 50% of the QC samples^54^. Peaks were detected and the metabolites could be left by using the interquartile range de-noising method. Half of the minimum value was used to fill missing values of raw data. Data analysis was accomplished using the internal standard normalization method. Peak numbers, sample names, and normalized peak areas of the three-dimensional data were then analyzed in principal component analysis (PCA) and orthogonal projections to latent structures-discriminate analysis (OPLS-DA) using the SIMCA14.1 software package (V14.1, MKS Data Analytics Solutions, Umea, Sweden). Principal component analysis (PCA) was first used to obtain an overview of group clustering and search for possible outliers. Subsequently, OPLS-DA was used to filter out the orthogonal variables that were not related to the classification variables. Further, OPLS-DA was used to analyze the non-orthogonal and orthogonal variables separately, which can lead to more reliable data and other relevant information regarding experimental groupings^55^. Model quality was validated on the basis of the following parameters: R^2^X (change in X explained by the model), R^2^Y (change in Y explained by the model), and Q^2^ (sum parameter in cross-validation)^56^. A loading plot was then constructed that was based on the OPLS-DA results in order to show the contribution of variables to differences between groups. The first principal component of the variable importance in the projection (VIP) was obtained to refine this analysis. Candidate differential metabolites were first selected based on VIP values exceeding 1.0. In the second step, a Student’s *t*-test (*P*-value > 0.05) was used to assess the remaining variables, and variables were discarded between two comparison groups. To identify metabolite pathways, metabolite databases were queried, including KEGG (http://www.genome.jp/kegg/) and NIST (http://www.nist.gov/index.html). Pathway analysis was conducted using the web-based Metabo Analyst tool that incorporates high-quality KEGG metabolic pathways as the backend knowledgebase.

All of the data (except the metabolomic data) are reported as means ± standard deviations and were assessed for significant statistical differences using independent *t* tests and one-way ANOVA in the IBM SPSS Statistics Version 22 software package. A *P* value < 0.05 was used to identify statistically significant associations.

## Supplementary information

Supplementary Figures and Tables.

Table S1

Table S3

Table S4

## Data Availability

The datasets generated and analyzed in the current study are available from the corresponding author on reasonable request.
