# Supplementary material for: Untargeted metabolomic analysis of the carotenoid-based orange coloration in Haliotis gigantea using GC-TOF-MS
Source: Sci Rep. 2019 Oct 10;9:14545. doi: 10.1038/s41598-019-51117-9 (PMC6787195; doi:10.1038/s41598-019-51117-9)
Supplement: Supplementary file 1 — Supplementary Figures and Tables. [file 41598_2019_51117_MOESM1_ESM.pdf]

Untargeted metabolomic analysis of the carotenoid-based orange coloration in *Haliotis gigantea* using GC-TOF-MS

Xiaohui Wei [1,2,3 #], Nan Chen [1,3 #], Bin Tang[1,2,3], Xuan Luo[3,4], Weiwei You [1,2,4 *], Caihuan Ke [1,2,4 *]

Figure legends:

Figure S1. Permutation test of OPLS-DA model for different groups: (a) H-F-S vs R-F-S, (b) H-M vs R-M, (c) H vs R, (d) H-F-S vs H-M, and (e) R-F-S vs R-M.

Figure S2. Loading plots derived from the OPLS-DA models for different groups: (a) H-F-S vs R-F-S, (b) H-M vs R-M, (c) H vs R, (d) H-F-S vs H-M, and (e) R-F-S vs R-M.

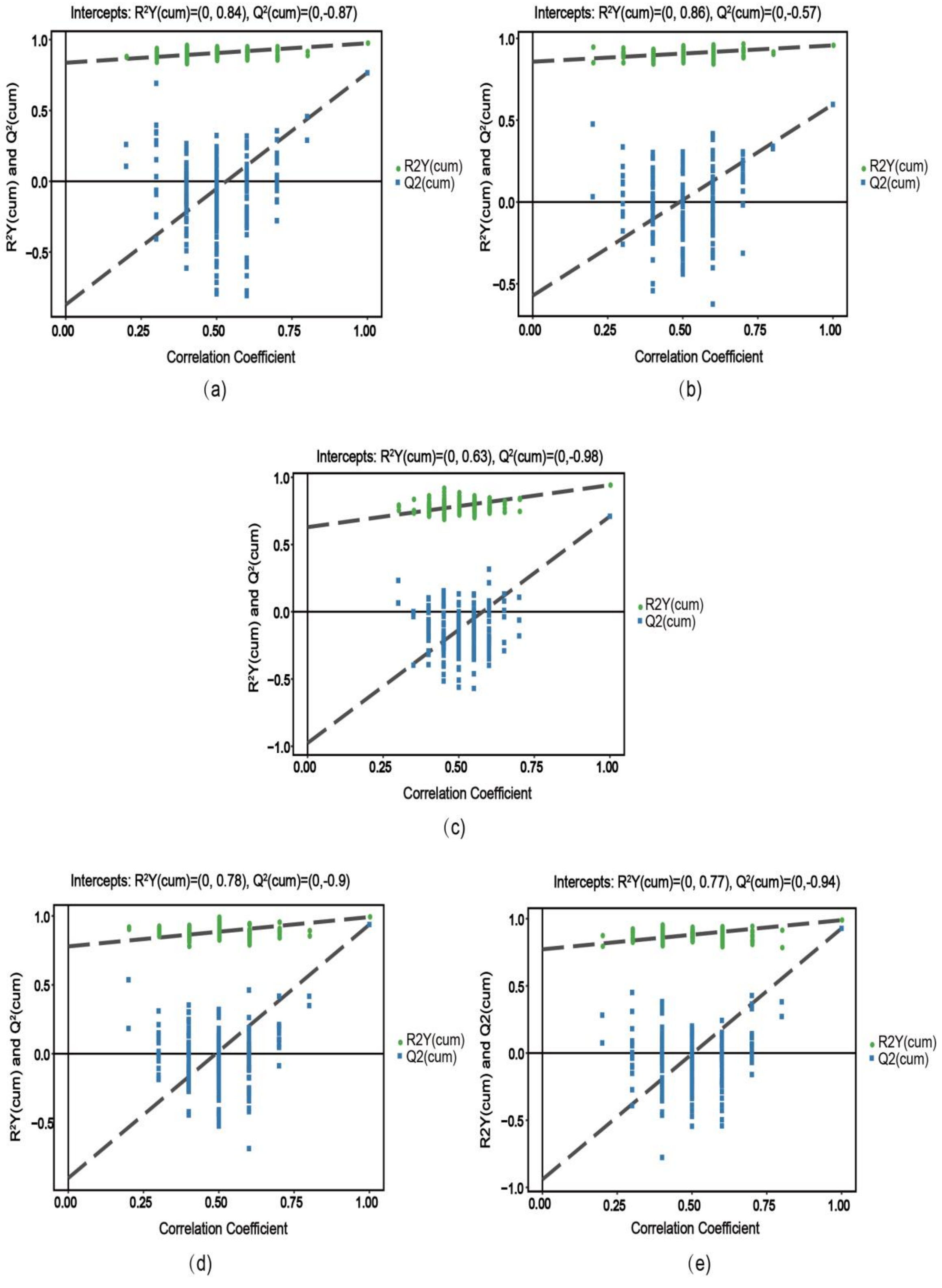

Intercepts: R²Y(cum)=(0, 0.84), Q²(cum)=(0,-0.87)
R²Y(cum) and Q²(cum)
Correlation Coefficient
R2Y(cum)
Q2(cum)
(a)
Intercepts: R²Y(cum)=(0, 0.86), Q²(cum)=(0,-0.57)
R²Y(cum) and Q²(cum)
Correlation Coefficient
R2Y(cum)
Q2(cum)
(b)
Intercepts: R²Y(cum)=(0, 0.63), Q²(cum)=(0,-0.98)
R²Y(cum) and Q²(cum)
Correlation Coefficient
R2Y(cum)
Q2(cum)
(c)
Intercepts: R²Y(cum)=(0, 0.78), Q²(cum)=(0,-0.9)
R²Y(cum) and Q²(cum)
Correlation Coefficient
R2Y(cum)
Q2(cum)
(d)
Intercepts: R²Y(cum)=(0, 0.77), Q²(cum)=(0,-0.94)
R2Y(cum) and Q2(cum)
Correlation Coefficient
R2Y(cum)
Q2(cum)
(e)

Figure S1

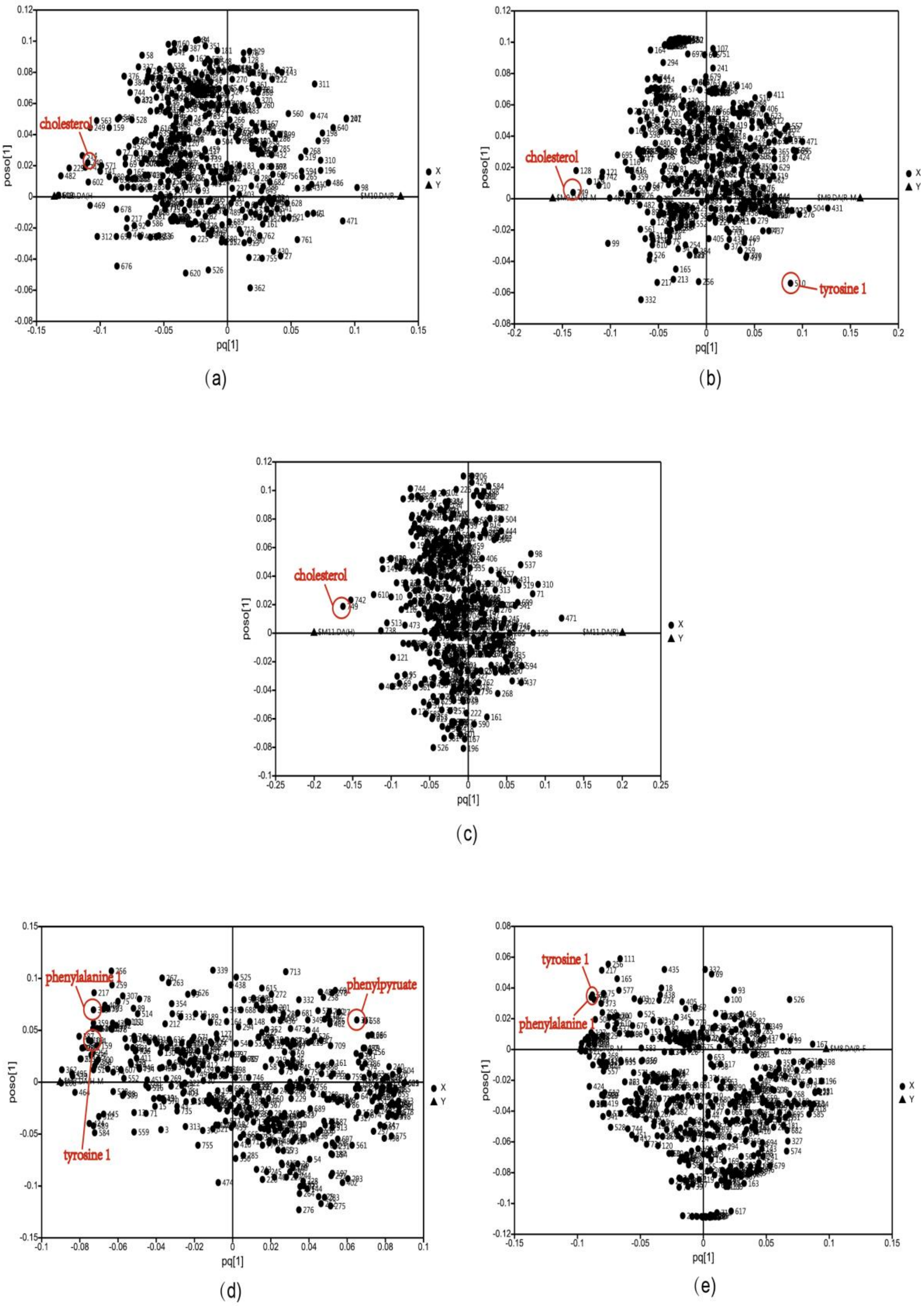

cholesterol
tyrosine 1
phenylalanine 1
phenylpyruate
poso[1]
pq[1]
X
Y
(a)
(b)
(c)
(d)
(e)

Figure S2

Table S2. Validation Parameters of OPLS-DA model

Title	Type	A	N	R^2X(cum)	R^2Y(cum)	Q^2 (cum)
**R-F-S vs H-F-S**	**OPLS-DA**	**1+1+0**	**20**	**0.298**	**0.974**	**0.766**
**R-M vs H-M**	**OPLS-DA**	**1+1+0**	**20**	**0.281**	**0.957**	**0.596**
**R vs H**	**OPLS-DA**	**1+1+0**	**40**	**0.176**	**0.942**	**0.71**
**H-F-S vs H-M**	**OPLS-DA**	**1+1+0**	**20**	**0.341**	**0.992**	**0.938**
**R-F-S vs R-M**	**OPLS-DA**	**1+1+0**	**20**	**0.405**	**0.989**	**0.928**

Title：the data of the model; Type：the model type of SIMCA；A：number of principal components in the model；N：number of observations in the model (this is the number of samples)；R^2X(cum)：represents the explanatory of the X variable for the model；R^2Y(cum)：represents the explanatory of the Y variable for the model；Q^2(cum)：predictability of the model.
